# Application of Entity-BERT model based on neuroscience and brain-like cognition in electronic medical record entity recognition

**DOI:** 10.3389/fnins.2023.1259652

**Published:** 2023-09-20

**Authors:** Weijia Lu, Jiehui Jiang, Yaxiang Shi, Xiaowei Zhong, Jun Gu, Lixia Huangfu, Ming Gong

**Affiliations:** ^1^Science and Technology Department, Affiliated Hospital of Nantong University, Nantong, China; ^2^Jianghai Hospital of Nantong Sutong Science and Technology Park, Nantong, China; ^3^Department of Biomedical Engineering, Shanghai University, Shanghai, China; ^4^Network Information Center, Zhongda Hospital Southeast University, Nanjing, China; ^5^School of Information and Control Engineering, China University of Mining and Technology, Xuzhou, China; ^6^Department of Respiratory, Affiliated Hospital Nantong University, Nantong, China; ^7^Information Center Department, Affiliated Hospital of Nantong University, Nantong, China

**Keywords:** BERT, LSTM, cross attention, entity recognition, electronic medical records

## Abstract

**Introduction:**

In the medical field, electronic medical records contain a large amount of textual information, and the unstructured nature of this information makes data extraction and analysis challenging. Therefore, automatic extraction of entity information from electronic medical records has become a significant issue in the healthcare domain.

**Methods:**

To address this problem, this paper proposes a deep learning-based entity information extraction model called Entity-BERT. The model aims to leverage the powerful feature extraction capabilities of deep learning and the pre-training language representation learning of BERT(Bidirectional Encoder Representations from Transformers), enabling it to automatically learn and recognize various entity types in medical electronic records, including medical terminologies, disease names, drug information, and more, providing more effective support for medical research and clinical practices. The Entity-BERT model utilizes a multi-layer neural network and cross-attention mechanism to process and fuse information at different levels and types, resembling the hierarchical and distributed processing of the human brain. Additionally, the model employs pre-trained language and sequence models to process and learn textual data, sharing similarities with the language processing and semantic understanding of the human brain. Furthermore, the Entity-BERT model can capture contextual information and long-term dependencies, combining the cross-attention mechanism to handle the complex and diverse language expressions in electronic medical records, resembling the information processing method of the human brain in many aspects. Additionally, exploring how to utilize competitive learning, adaptive regulation, and synaptic plasticity to optimize the model's prediction results, automatically adjust its parameters, and achieve adaptive learning and dynamic adjustments from the perspective of neuroscience and brain-like cognition is of interest.

**Results and discussion:**

Experimental results demonstrate that the Entity-BERT model achieves outstanding performance in entity recognition tasks within electronic medical records, surpassing other existing entity recognition models. This research not only provides more efficient and accurate natural language processing technology for the medical and health field but also introduces new ideas and directions for the design and optimization of deep learning models.

## 1. Introduction

With the advent of the digital age, a large amount of medical data, including electronic medical records (EMRs) (Yu et al., 2019), has been digitized. EMRs serve as a crucial source of information for doctors in making diagnostic and treatment decisions, but their unstructured nature makes information extraction and analysis challenging (Li et al., 2020). Extracting valuable entity information from EMRs remains a challenging task. Many researchers have proposed various deep learning models to automatically extract entity information from EMRs (Wang et al., 2019). The successful application of these models can provide doctors with faster and more accurate insights into patients' conditions, aiding them in making better diagnostic and treatment decisions, thereby enhancing the quality of medical care and patient survival rates. The following are five commonly used models for extracting entities from EMRs.

1. CRF Model (Dai et al., 2019): The Conditional Random Fields (CRF) model is a sequence labeling method based on probabilistic graphical models that has been widely used in the field of natural language processing. It can automatically learn the probability distribution of labeled sequences and use these probabilities to predict labeled sequences. The advantage is that it can handle complex labeled sequences, but the disadvantage is that it requires manual feature design and cannot handle long-term dependencies.
2. LSTM-CRF Model (Tang et al., 2019): The LSTM-CRF model adds an LSTM neural network to the CRF model to capture long-term dependencies. This model can automatically learn features and can handle long-term dependencies, but requires a large amount of training data.
3. BiLSTM-CRF Model (Jiang et al., 2019): The BiLSTM-CRF model adds a bidirectional LSTM neural network to the LSTM-CRF model to capture contextual information. This model can handle more complex language structures and contextual information, but requires more computational resources.
4. Transformer-CRF Model (Zhang et al., 2021): The Transformer-CRF model adds a Transformer neural network to the CRF model to capture contextual information. Compared with LSTM and BiLSTM, the Transformer-CRF model has better parallelism and faster training speed.
5. BERT-CRF Model (Grancharova and Dalianis, 2021): The BERT-CRF model is a deep learning model based on BERT and CRF that uses BERT to encode input text and CRF to perform entity recognition. The BERT-CRF model can use the contextual information of BERT to better capture the language structure of the input text and automatically learn the probability distribution of labeled sequences.

All of the above commonly used models require a large amount of training data and computational resources. Therefore, to address this issue and extract important information from EMRs, this paper proposes an Entity Recognition in Electronic Medical Records Based on BERT and LSTM Model With Cross Attention (Entity-BERT) to process this issue. The Entity-BERT model first uses BERT to encode the input text and generate contextualized word embeddings. The output of BERT is then input to a bidirectional LSTM layer to capture the sequence dependencies between words. Then, using the cross-attention mechanism, the model focuses on relevant text parts when making predictions. This model has the ability to capture contextual information and long-term dependencies, and combined with the cross-attention mechanism, makes it very suitable for handling the complex and variable language in EMRs.

- The Entity-BERT model combines the BERT and LSTM networks to simultaneously capture contextual information and long-term dependencies, thereby better extracting entity information from electronic medical records.
- The model introduces a cross-attention mechanism, allowing the model to focus on relevant text parts when making predictions and improving the accuracy and generalization performance of the model.
- Experimental validation was conducted on two publicly available datasets, demonstrating that the Entity-BERT model outperforms other commonly used models in extracting entity information from electronic medical records. This confirms the effectiveness and practicality of the Entity-BERT model.

In the rest of this paper, we will present recent related work in Section 2. Section 3 introduces an overview of our proposed methods, including the BERT model and LSTM model. Section 4 showcases the experimental part, which includes detailed experimental design and comparative experiments. Finally, Section 5 provides the conclusion.

## 2. Related work

Currently, research on automatically extracting entity information from electronic medical records is mainly manifested in natural language processing (NLP) techniques, deep learning models, and conditional random fields (CRF) models. These models have made significant advancements in data extraction and analysis in the medical field.

### 2.1. NLP

With the digitization of electronic medical records and other healthcare data, natural language processing (NLP) (Dai et al., 2019) technology is increasingly being applied in the medical field. Among these applications, NLP in electronic medical records has received particular attention. Electronic medical records are an electronic form of recording patient diagnosis and treatment information by hospitals and other medical institutions, which contains a large amount of medical terminology and specialized knowledge. NLP technology can help doctors better manage and utilize this data (Torres Cabán et al., 2022).

The main applications of NLP in electronic medical records include the following aspects: Entity extraction: Entity extraction is an important application of NLP in electronic medical records (Gligic et al., 2020). Electronic medical records contain a vast amount of medical terminology and domain-specific knowledge. Entity extraction can extract these medical terms and knowledge from the records, helping doctors better understand and analyze patients' diagnostic and treatment information. For example, entity extraction can identify entities such as symptoms, drugs, and diseases in electronic medical records, assisting doctors in comprehending patients' conditions and formulating treatment plans. Named entity recognition: Named entity recognition is the task of identifying medical terms and domain-specific knowledge in electronic medical records. This process involves extracting these terms and knowledge to aid doctors in understanding patients' conditions and devising treatment plans (Papadaki et al., 2022). The study found that the BERT-BiLSTM-CRF model achieved an F1 score of approximately 75%. Relationship extraction: Relationship extraction involves identifying relationships between medical terms and domain-specific knowledge in electronic medical records. By extracting these relationships, doctors can better understand patients' conditions and formulate treatment plans. Text classification (Santiso et al., 2020): Text classification is the task of categorizing text in electronic medical records into different classes. Since electronic medical records contain a vast amount of medical information, text classification can help categorize this information into relevant classes, allowing doctors to better understand patients' conditions and devise treatment plans. The study reported an accuracy of 65.1 for exact matches and 82.4 for partial matches in a Spanish electronic health record dataset. Question-answering system (Vinod et al., 2021): The question-answering system can respond to medical questions posed by doctors and patients. By leveraging the medical terms and domain-specific knowledge in electronic medical records, the system can answer medical queries, assisting doctors and patients in gaining better insights into patients' conditions and treatment options. The research achieved significant results in biomedical question answering (English) with strict accuracies (S) of 27.78, 42.61, and 42.38 for the BioASQ 4b, BioASQ 5b, and BioASQ 6b datasets, respectively.

### 2.2. Deep learning model

Currently, there are still many challenges in the processing of electronic medical records in the medical field. For example, Wireless Capsule Endoscopy (WCE) devices in medical applications typically generate over 60,000 images during gastrointestinal procedures. These images need to be examined by a professional doctor in an attempt to identify frames that may contain inflammation or diseases (Zou et al., 2022a). For a physician, going through such a large number of examinations is very time-consuming, necessitating the urgent need for new methods to address this issue (Rahim et al., 2020). Entity extraction is an important application of deep learning in electronic medical record processing (Cai et al., 2019). It can automatically extract entities such as symptoms, drugs, and diseases from electronic medical records, helping doctors better understand and analyze patient diagnosis and treatment information. Entity extraction has wide-ranging applications in the medical field, including disease diagnosis, drug therapy, and clinical research (Gligic et al., 2020). For instance, recent research on electromyography (EMG) records in bio-signals and the capture of mixed signals on electrodes cannot be directly observed through non-invasive methods, which has been a challenging issue. Researchers have proposed a solution called the cyclic force meter, which implements a treatment machine's fuzzy speed controller. Through various experimental tests, the effectiveness of the designed controller has been verified, greatly improving the system's robustness (Ensastiga et al., 2022). Additionally, the biomedical engineering task of classifying motion-corresponding EMG signals has received extensive attention. Researchers have employed a Multi-Layer Perceptron (MLP) for EMG signal processing, which has proven effective in signal processing and pattern classification. By optimizing four important hyperparameters, the model's performance was enhanced, achieving an average classification rate of 93% in the validation stage (Aviles et al., 2023). Furthermore, researchers have utilized the Medical Visual Language BERT (Medical-vLBERT) model to identify abnormalities on 2019 coronavirus disease scans and automatically generate medical reports based on the detected lesion areas. The results demonstrated state-of-the-art performance in terms prediction and report generation (Ning et al., 2023). This also indicates the widespread use of deep learning models in entity extraction research. Here, are several common deep learning models.

Convolutional neural networks (CNNs) are commonly used models in deep learning. They can extract feature information from input data through convolution and pooling operations. In entity extraction, CNNs can be employed to extract medical entities from electronic medical records. For example, Yin et al. (2019) proposed a CNN-based model for extracting relations between medical terms and specialized knowledge from electronic medical records. Experimental results demonstrated that the model achieved F1 scores of 93.00 and 86.34% on the CCKS-2017 and TP_CNER datasets, respectively. However, CNNs also have some limitations, especially in the field of medical imaging. Obtaining comprehensive annotated datasets like ImageNet remains a challenge in medical imaging tasks. To address this issue, several main techniques have been successfully used for CNN-based medical image classification: training CNN from scratch, using pre-trained CNN features, unsupervised CNN pre-training followed by supervised fine-tuning, and transfer learning by fine-tuning a CNN model pre-trained on natural image datasets for medical image tasks. In conclusion, CNNs play a crucial role as important models in deep learning for tasks such as entity extraction and medical image classification (Zou et al., 2022b). Nonetheless, continuous exploration and optimization are still required to fully unleash their potential and overcome potential challenges, especially in the medical domain.

Recurrent neural networks (RNNs) are commonly used models in deep learning. They can model input data sequences to capture their time-series information. In entity extraction, RNNs can be used to extract medical entities from electronic medical records. For example, Gligic et al. (2020) proposed an RNN-based model for extracting symptoms and drugs from electronic medical records. Experimental results showed that the model achieved an F1 score of 82.4%.

Deep learning model ensembles are commonly used methods in deep learning. They can combine multiple types of deep learning models to improve performance and effectiveness. In entity extraction, deep learning model ensembles can be used to extract medical entities from electronic medical records. For example, Li et al. (2019) proposed a Transformer-based model for extracting medical entities from electronic medical records. Experimental results showed that the model achieved better performance than other widely used models without additional features (F1-measure of 85.4% for CCKS 2018 and 90.29% for CCKS 2017), and it performed best in terms of POS and dictionary features (F1-measure of 86.11% for CCKS 2018 and 90.48% for CCKS 2017).

### 2.3. Conditional random fields model

The CRF model is a sequence labeling method based on probabilistic graphical models, which is widely used in entity recognition tasks. In electronic medical record processing, entity recognition is an important task because it can automatically extract entities such as symptoms, drugs, and diseases from electronic medical records, helping doctors better understand and analyze the diagnosis and treatment of patients (Wu et al., 2023). In entity recognition tasks in electronic medical records, CRF models are often used to label medical terms, symptoms, drugs, diseases, and other entities. The CRF model can label each word in the input text as an entity or non-entity, and adjust parameters based on training data to achieve entity recognition. In entity recognition tasks, the CRF model can handle complex labeling sequences, such as labeling entities as different categories. In electronic medical record processing, the CRF model is widely used in entity recognition tasks. For example, Lin and Xie (2020) proposed a CRF-based model for extracting symptoms and drug entities from Chinese electronic medical records. The model used bidirectional LSTM as a feature extractor and CRF for labeling. Experimental results showed that the model achieved good performance in entity recognition tasks.

In addition, the CRF model can be combined with other deep learning models, such as LSTM, CNN, etc. For example, Lin and Xie (2020) proposed an LSTM-CRF model for extracting symptoms from electronic medical records. The model used LSTM for feature extraction and CRF for labeling. Experimental results showed that the model achieved good performance in entity recognition tasks. The CRF model is a foundational model for entity recognition tasks in electronic medical record processing and is widely used in practice. Although it has some limitations, such as its inability to handle long-term dependencies, it provides a foundation and benchmark for more advanced models. In practical applications, the appropriate model can be selected based on the specific task and combined with other techniques for improvement and optimization.

## 3. Methodology

Below, we will introduce the Entity-BERT model, the BERT model, and the LSTM model, respectively. These models have made significant contributions to extracting medical entities from electronic medical records, converting input medical text into meaningful word embeddings, and analyzing the temporal relationships in medical sequences. They provide valuable insights for research on electronic medical record processing and other natural language processing tasks.

### 3.1. Overview of our network

**Figure 1:**
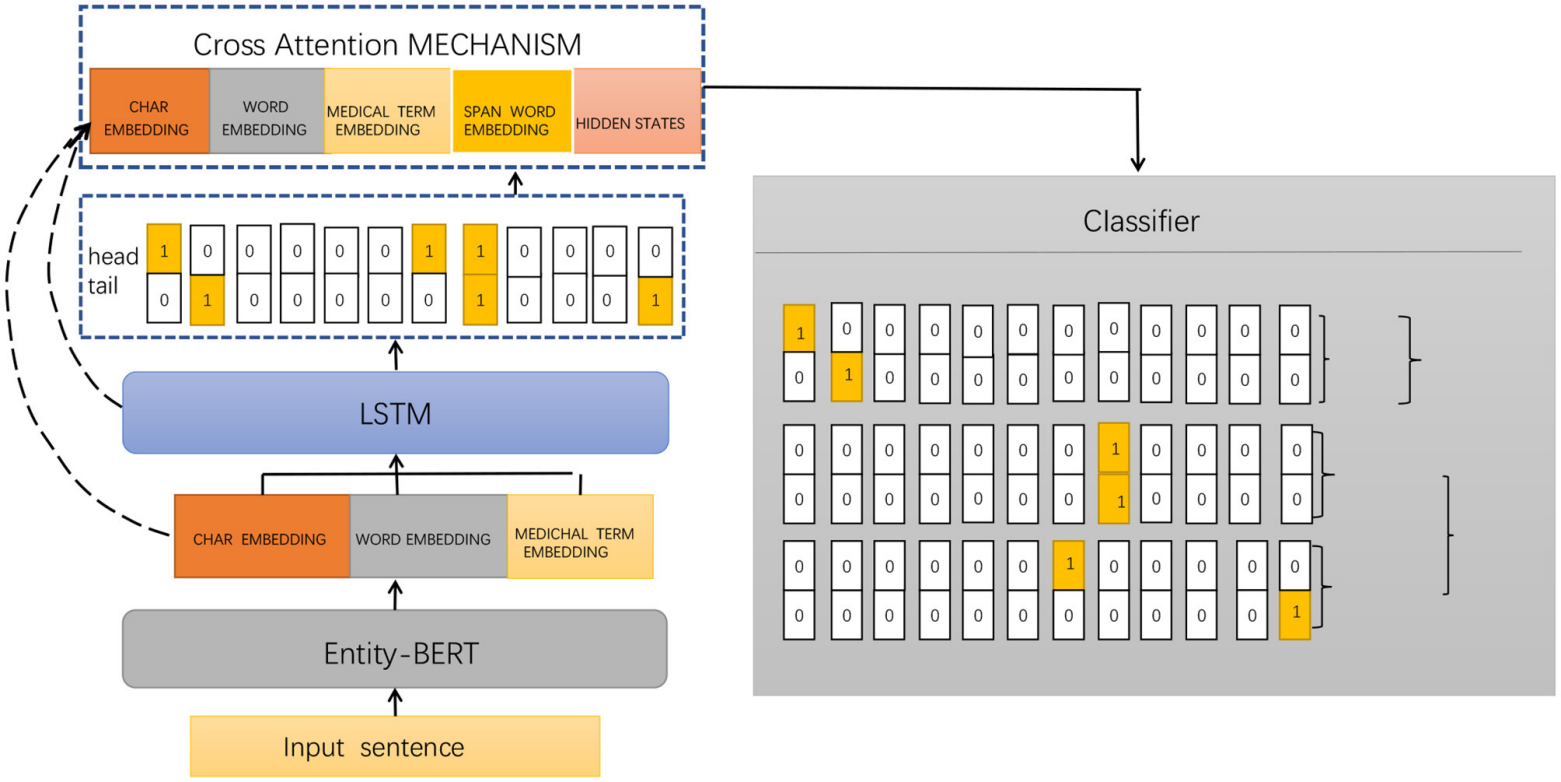
Framework diagram of our proposed method.

Entity-BERT is an entity recognition method based on BERT and LSTM models, which uses deep learning techniques to automatically extract medical terms, symptoms, drugs, diseases, and other entities from electronic medical records. This helps doctors better understand and analyze the diagnosis and treatment of patients. The method is mainly composed of a BERT model, an LSTM model, and a cross-attention mechanism, which can better handle the associated information between entities and has high accuracy and recall rates. Figure 1 shows the framework of the proposed method.

The implementation process of Entity-BERT mainly includes the following steps:

1. Data preprocessing: In the data preprocessing stage, the electronic medical record data needs to be cleaned and labeled, and entity tags need to be added to the text. The cleaning process includes removing useless information, removing punctuation, and converting case, etc. The labeling process requires assigning corresponding labels to each entity in the text, such as symptoms, drugs, diseases, etc. Existing labeling tools can be used or self-developed labeling programs can be used to complete this step.
2. BERT encoding: BERT is a pre-trained language model that can encode text and obtain context-related word vector representations. In Entity-BERT, a pre-trained BERT model can be used to encode electronic medical record text and obtain context-related word vector representations. Specifically, the output of the BERT model can be used to concatenate the word vector representations of each word, obtaining the word vector representation of the entire sentence.
3. Feature extraction: In the feature extraction stage, an LSTM model can be used as a feature extractor to process the encoded text and obtain richer and more abstract feature representations. Specifically, the output of the BERT model can be used as the input of the LSTM model. The LSTM model can process the input sequence step by step according to time steps and use the output of each time step as the input of the subsequent time step. This way, more complex and richer feature representations can be obtained, which is beneficial to improve the performance of the model.
4. Entity recognition: In the entity recognition stage, a cross-attention mechanism can be used to capture the associated information in the input sequence and improve the model's ability to recognize entities. Specifically, the output vectors of the LSTM model and the BERT model can be concatenated, and the cross-attention mechanism can be used to weight them, obtaining context-related feature representations. Then, a softmax classifier can be used to classify each word and identify the entity.
5. Model evaluation: In the model evaluation stage, multiple electronic medical record datasets are used to evaluate the model, and performance metrics such as accuracy, recall, and F1 score are used to evaluate the performance and effectiveness of the model. If the model's performance is not satisfactory, methods such as adjusting the model structure and adjusting hyperparameters can be tried to improve its performance.

The implementation process of Entity-BERT includes data preprocessing, BERT encoding, feature extraction, entity recognition, and model evaluation. By applying deep learning techniques, the automatic recognition of entities in electronic medical records can be achieved, which improves the efficiency and accuracy of doctors' diagnosis and treatment and provides a reference for the research on electronic medical record processing and other natural language processing tasks.

### 3.2. BERT model

**Figure 2:**
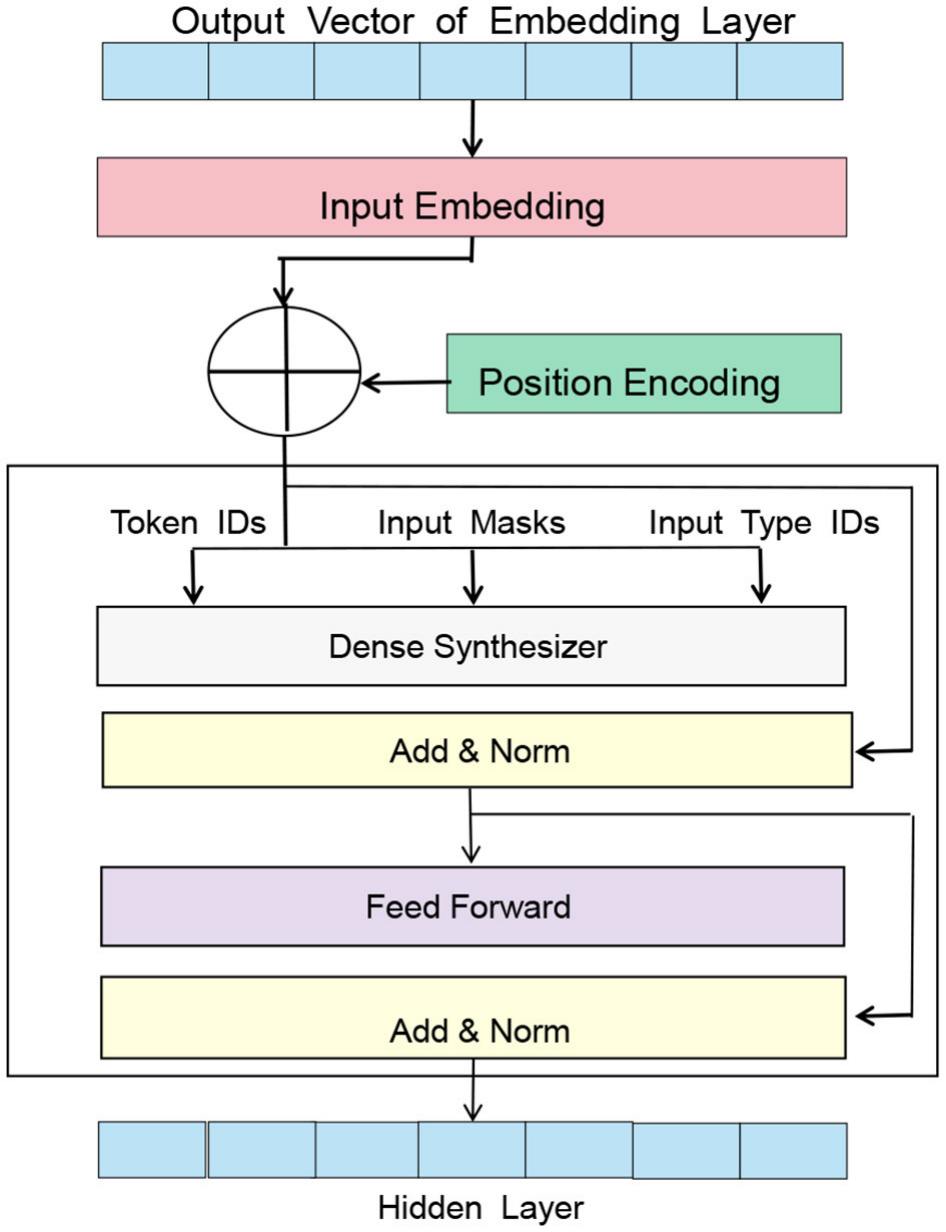
A schematic diagram of the BERT model.

BERT (Dai et al., 2019), short for Bidirectional Encoder Representations from Transformers, is a pre-trained language model proposed by Google in 2018. Based on the Transformer architecture, it can perform unsupervised pre-training on large-scale corpora to generate high-quality language representations. Figure 2 is a schematic diagram of the BERT model.

The basic principle of the BERT model is to learn context-related word vector representations from large-scale text data in an unsupervised manner, where the representation of each word in the context is related to the surrounding words (Kim and Lee, 2020), thus better capturing the meaning and context of words. The pre-training tasks of the BERT model include Masked Language Model and Next Sentence Prediction.

In the Entity-BERT method, the role of the BERT model is to convert the input electronic medical record text into corresponding word vector representations while preserving context-related information. Specifically, in the data preprocessing stage, the electronic medical record data needs to be cleaned and labeled, and the labeled text is input to the BERT model for encoding. The output of the BERT model is the context-related word vector representation of each word, which includes the meaning of the word and its semantic information in the context.

In the feature extraction stage, the output of the BERT model is used as the input of the LSTM model, which can further extract features and model the temporal relationship of the sequence. Since the encoding result of the BERT model has already considered the context information, the LSTM model can more accurately extract the temporal information of the sequence, further improving the performance of the model.

In the entity recognition stage, the output of the BERT model is used to perform cross-attention calculation with the output of the LSTM model, capturing the associated information in the input sequence and improving the model's ability to recognize entities. The encoding result of the BERT model retains the context information of the input sequence, which can help the model better understand the entity labels in the input sequence, thus improving the accuracy and recall rate of entity recognition. The BERT model plays an important role in the Entity-BERT method, encoding the input text and preserving context-related information, providing crucial semantic information for subsequent feature extraction and entity recognition.

The formula of the BERT model is as follows:


(1) $$\begin{array}{l} h_{i}=f\left(\overset{n}{\underset{j=1}{\sum }}\alpha_{i,j}g\left(W_{h}^{\left(h\right)}h_{j}+W_{h}^{\left(e\right)}e_{j}+b_{h}\right)\right) \end{array}$$

Among them, *h*_*i*_ represents the word vector representation of the *i*th word, *g* is a non-linear activation function, *f* is a linear transformation, *e*_*j*_ is the embedding vector of the *j*th word, $W_{h}^{\left(h\right)}$, $W_{h}^{\left(e\right)}$ and *b*_*h*_ are learnable parameters. α_*i, j*_ is the attention weight used to calculate the relative importance between the *i*th word and the *j*th word. The attention weight is calculated as follows:


(2) $$\begin{array}{l} \alpha_{i,j}=\frac{\exp\left(e_{i,j}\right)}{\sum_{k=1}^{n}\exp\left(e_{i,k}\right)} \end{array}$$

Among them, *e*_*i,j*_ is the similarity score between the *i*th word and the *j*th word, calculated by the following formula:


(3) $$\begin{array}{l} e_{i,j}=\text{Attention}\left(h_{i},h_{j}\right)=\frac{{\left(W_{a}h_{i}\right)}^{T}\left(W_{a}h_{j}\right)}{\sqrt{d}} \end{array}$$

Among them, *W*_*a*_ is a learnable parameter, and *d* is the dimension of the word vector.

The training of the BERT model is divided into two stages. The first stage is the pre-training stage, which uses a large-scale unlabeled corpus for training. The goal is to learn context-dependent word vector representations. The second stage is the fine-tuning stage, which uses labeled task data for fine-tuning to adapt to specific tasks.

### 3.3. LSTM model

**Figure 3:**
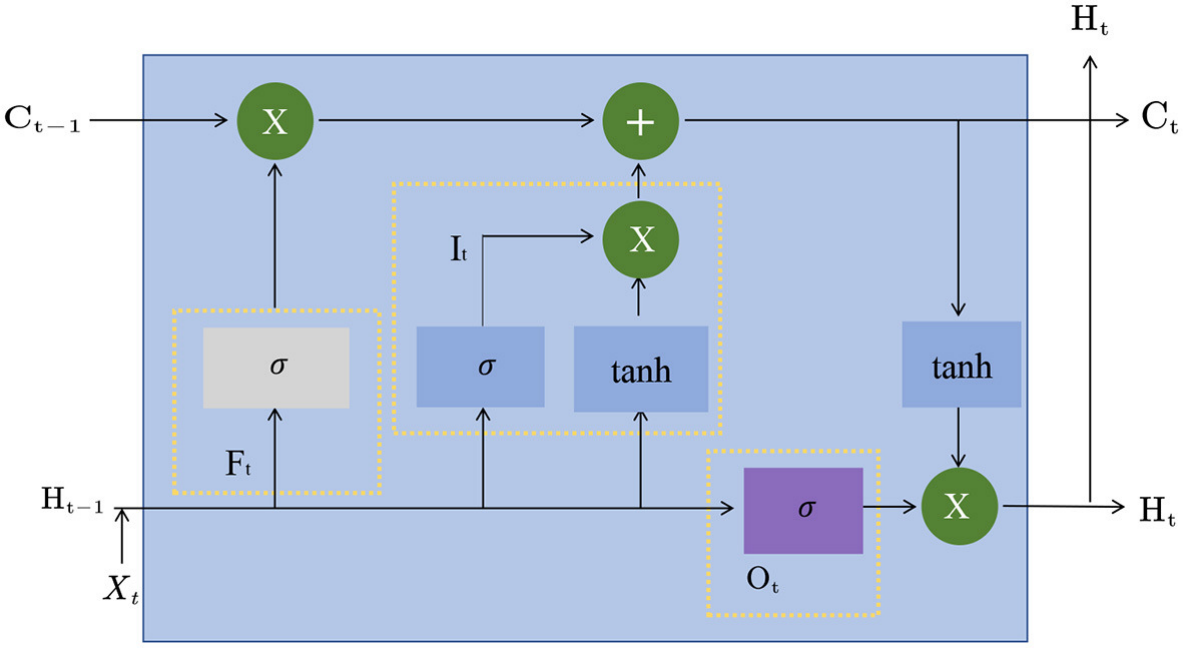
A schematic diagram of the principle of LSTM.

LSTM, or Long Short-Term Memory, is a Recurrent Neural Network model commonly used (Ji et al., 2019). Compared with the traditional cyclic neural network, the LSTM model can better preserve and utilize historical information when processing long sequences, avoiding the problem of gradient disappearance or gradient explosion. Figure 3 is a schematic diagram of the principle of LSTM.

The basic principle of the LSTM model is to introduce three gating structures, namely the input gate, output gate, and forget gate. The input gate controls whether the newly input information is added to the cell state (Dong et al., 2019), the output gate controls whether the information in the cell state is output, and the forget gate controls whether the information in the cell state is forgotten. These gating structures can be learned to adapt to different tasks and data.

In the LSTM model, the input of each time step is the input of the current time step and the hidden state of the previous time step, and the output is the hidden state of the current time step and the cell state. The formula of the LSTM model is as follows:


(4) $$\begin{array}{l} i_{t}=\sigma \left(W_{xi}x_{t}+W_{hi}h_{t-1}+b_{i}\right) \end{array}$$

(5) $$\begin{array}{l} f_{t}=\sigma \left(W_{xf}x_{t}+W_{hf}h_{t-1}+b_{f}\right) \end{array}$$

(6) $$\begin{array}{l} o_{t}=\sigma \left(W_{xo}x_{t}+W_{ho}h_{t-1}+b_{o}\right) \end{array}$$

(7) $$\begin{array}{l} c_{t}=f_{t}c_{t-1}+i_{t}\tanh\left(W_{xc}x_{t}+W_{hc}h_{t-1}+b_{c}\right) \end{array}$$

(8) $$\begin{array}{l} h_{t}=o_{t}\tanh\left(c_{t}\right) \end{array}$$

Among them, *x*_*t*_ is the input of the current time step, *h*_*t*−1_ is the hidden state of the previous time step, *i*_*t*_, *f*_*t*_, and *o*_*t*_ represent the input gate, forget gate, and output gate, respectively. The output value of, *c*_*t*_ represents the cell state of the current time step, σ represents the Sigmoid function, tanh represents the hyperbolic tangent function, *W* and *b* are learnable parameters.

In the Entity-BERT method, the role of the LSTM model is to extract features further and model the temporal relationship of the sequence. Specifically, in the feature extraction stage, the output of the BERT model is used as the input of the LSTM model, which can further extract features and model the temporal relationship of the sequence. Since the encoding result of the BERT model has considered the context information, the LSTM model can more accurately extract the timing information of the sequence, further improving the model's performance. In the entity recognition stage, the output of the LSTM model is used to perform cross-attention calculations with the output of the BERT model, thereby capturing the associated information in the input sequence and improving the model's ability to recognize entities. The LSTM model plays an important role in the Entity-BERT method, further improving the model's performance by extracting the temporal relationship of the sequence and capturing the associated information in the input sequence.

### 3.4. Cross attention mechanism

**Figure 4:**
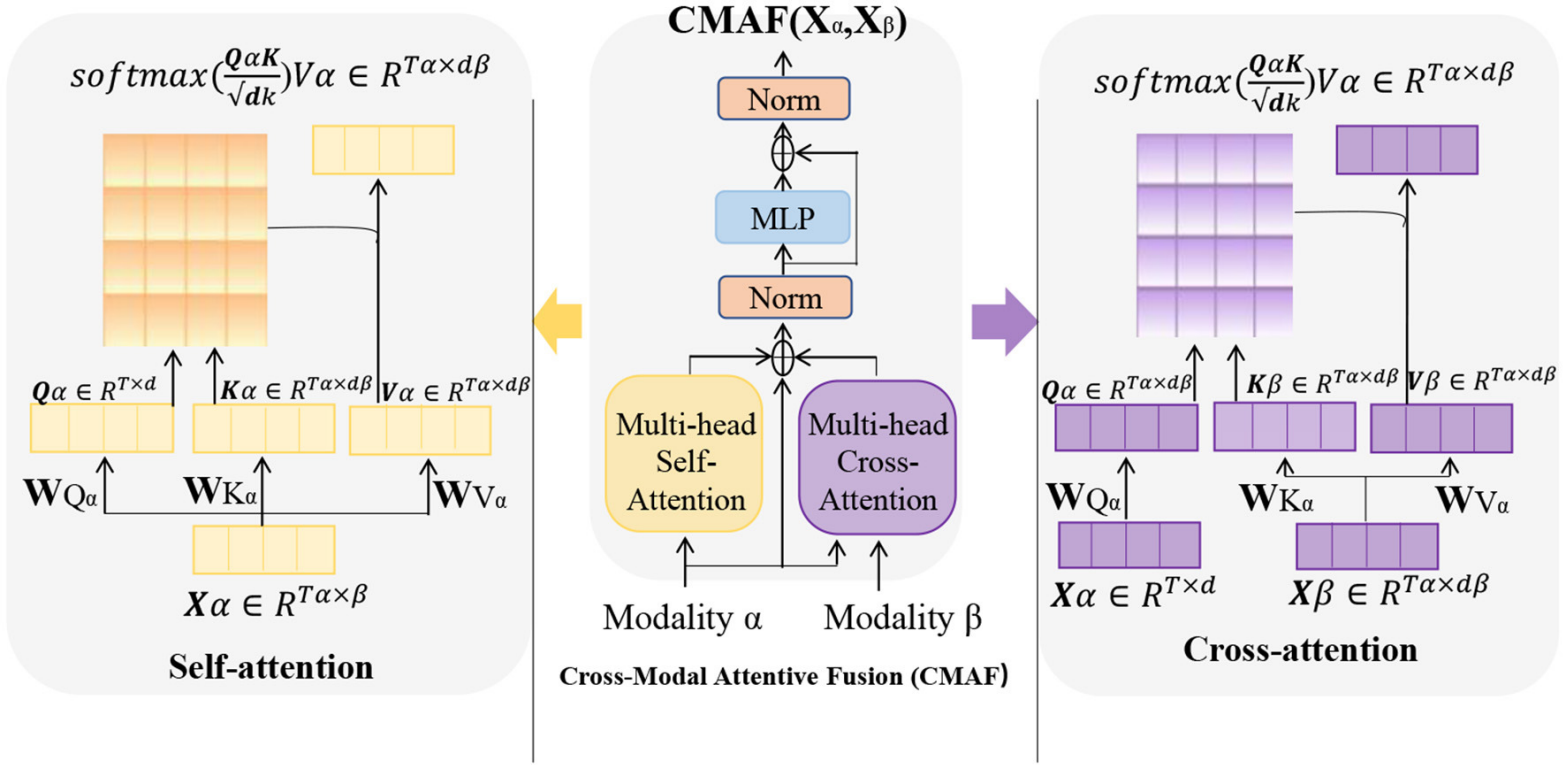
A schematic diagram of the Cross Attention mechanism. Where, the parameters *W*_*qa*_, *W*_*ka*_, and *W*_*va*_ are weight matrices used to perform linear transformations on the input variable *X*. The vector *Q* (“Query”) is the query vector, used to measure the importance of each position in the input variable. The vector *K* (“Key”) represents the key vector, which captures the features of each position in the input variable. The vector *V* (“Value”) is used to represent the specific information at each position in the input variable.

Cross Attention is an attention mechanism used to calculate the interaction representation between two sequences (Kong et al., 2021). It can be used in many natural language processing tasks, such as machine translation, question answering systems, and text classification. In these tasks, attention mechanisms can help models better understand key information in the input sequence, thereby improving the performance of the model. Figure 4 is a schematic diagram of the Cross Attention mechanism.

The basic principle of Cross Attention is to calculate the similarity score between each position in input sequence A and all positions in sequence B. These scores are used to weight the various positions in sequence B, resulting in a weighted representation of sequence B, which is used to represent the interaction between each position in sequence A and sequence B. Similarly, the interaction representation of sequence A can also be calculated on sequence B.

In practical applications, Cross Attention is typically used in conjunction with other neural network structures. In natural language processing tasks, Cross Attention is often used with Transformer models (Yu et al., 2019). In Transformers, Cross Attention is used to calculate the interaction representation between the encoder and decoder, so that the input sequence information can be better utilized when generating the output sequence.

In the Entity-BERT model, Cross Attention is used to establish an interaction representation between BERT and LSTM. In this model, BERT and LSTM calculate different representations of the input text, and Cross Attention is used to calculate the interaction representation between them, so as to better utilize the input text information in entity recognition tasks.

Cross Attention Mechanism is a deep learning model used for processing multimodal inputs, primarily applied to tasks that involve interaction between vision and language, such as image captioning and visual question answering (Hao and Cao, 2020). The basic principle is to use attention mechanisms to achieve information interaction between different modalities, thereby improving the performance of the model.

In Cross Attention Mechanism, there are usually two inputs: one is the image input, and the other is the text input. Let the feature representation of the image input be **I** ∈ ℝ^*h*×*w*×*d*^, where *h*, *w*, and *d* represent the height, width, and feature dimension of the image, respectively. Let the feature representation of the text input be **T** ∈ ℝ^*l*×*e*^, where *l* represents the length of the text and *e* represents the word embedding dimension.

First, we need to calculate the similarity matrix **S**∈ℝ^*l*×*h*×*w*^ between the image input and the text input, where the element *s*_*ijk*_ represents the similarity between the *i*-th word in the text and the feature representation of position (*j, k*) in the image. Similarity can be calculated using dot product, bilinear, or multi-layer perceptron methods, among which bilinear is a commonly used method. Specifically, the bilinear method maps the feature representations of the image and text to the same low-dimensional space, and then calculates their dot product:


(9) $$\begin{array}{l} s_{ijk}={\text{T}}_{i}\text{W}a\text{I}jk \end{array}$$

where ${\text{W}}_{a}\in {\mathbb{R}}^{e\times d}$ is a learnable parameter matrix.

Then, we obtain the attention distributions **A**∈ℝ^*l*×*h*×*w*^ and **B**∈ℝ^*h*×*w*×*l*^ for the image-to-text and text-to-image directions, respectively, by normalizing the rows and columns of the similarity matrix **S**:


(10) $$\begin{array}{l} a_{ijk}=\frac{\exp\left(s_{ijk}\right)}{\overset{l}{\underset{i^{\prime }=1}{\sum }}\exp\left(s_{i^{\prime }jk}\right)},\;b_{jkl}=\frac{\exp\left(s_{klj}\right)}{\overset{h}{\underset{j^{\prime }=1}{\sum }}\overset{w}{\underset{k^{\prime }=1}{\sum }}\exp\left(s_{k^{\prime }lj^{\prime }}\right)} \end{array}$$

where *a*_*ijk*_ represents the attention distribution of the *i*-th word in the text for position (*j, k*) in the image, and *b*_*jkl*_ represents the attention distribution of position (*j, k*) in the image for the *l*-th word in the text. It can be seen that the calculation of attention distributions is based on the similarity matrix, which allows the model to focus on important features more accurately during information interaction.

Finally, we obtain the representation of the cross attention by taking a weighted sum of the image and text inputs:


(11) $$\begin{array}{l} \text{V}=\overset{l}{\underset{i=1}{\sum }}\overset{h}{\underset{j=1}{\sum }}\overset{w}{\underset{k=1}{\sum }}a_{ijk}\text{I}jk+\sum {j=1}^{h}\overset{w}{\underset{k=1}{\sum }}\overset{l}{\underset{l=1}{\sum }}b_{jkl}{\text{T}}_{l} \end{array}$$

where **V**∈ℝ^*d*^ represents the representation of the cross attention. It can be seen that the representation of the cross attention is a weighted sum of the image and text inputs, where the weights are obtained by calculating the attention distributions. This allows the fusion of information from both image and text, thereby improving the performance of the model.

## 4. Experiment

Below, we first provide a description of the datasets required for the experiments, followed by a detailed explanation of the experimental setup and details, including data preprocessing, model selection, and the training process. Additionally, we introduce the evaluation metrics for the models.

### 4.1. Datasets

In this paper, ACE 2004 (Doddington et al., 2004), ACE 2005 (Bentivogli et al., 2010), GENIA (Kim et al., 2003), KBP2017 (Ji et al., 2017) are selected for experiments.

ACE (Automatic Content Extraction) dataset is an English dataset widely used for information extraction and text classification tasks. Its task is to identify and extract a series of entities, relationships, events and other information from news articles. The ACE dataset was originally funded by the Defense Advanced Research Projects Agency (DARPA) of the US Department of Defense, with the aim of providing technical support for national security and counter-terrorism. After years of development and improvement, the ACE dataset has become one of the important benchmark test sets in the field of information extraction.

The ACE 2004 dataset (Doddington et al., 2004) contains articles from different news agencies, covering topics such as terrorism, crime, weapons, etc. These articles contain many entities, such as people, organizations, locations, dates, etc., as well as their relationships and events. The main tasks in the ACE 2004 dataset are entity recognition and relation extraction. The goal of entity recognition is to identify different types of entities, such as people, organizations, locations, dates, etc., from the text. The goal of relation extraction is to establish semantic relationships between the identified entities, such as relationships between people, organizations and locations, etc. These tasks have important applications in national security, intelligence analysis, knowledge graphs and other fields.

ACE 2005 (Bentivogli et al., 2010) is a follow-up version of ACE 2004, and is also an English dataset widely used for information extraction and text classification tasks. Unlike ACE 2004, the tasks in the ACE 2005 dataset are more complex, including event extraction, referential resolution, and time recognition. The goal of event extraction is to extract events from the text and identify information such as participants, time, and location. The goal of referential resolution is to determine the specific entities referred to by pronouns, noun phrases, etc. in the text. The goal of time recognition is to identify different types of time expressions, such as dates, time periods, time points, etc. These tasks have important implications for information extraction, natural language processing, and other fields.

In order to better evaluate the performance of algorithms and models, the ACE dataset provides strict evaluation standards and benchmark tests, including evaluation metrics, evaluation data, and so on. In recent years, many researchers and teams have conducted a lot of research work on the ACE dataset, proposing many entity recognition, relation extraction, and event extraction algorithms based on machine learning, deep learning and other methods, achieving good results. The continuous improvement and update of the ACE dataset will provide more challenging tasks and data for research and practice in the fields of information extraction and natural language processing, promoting the development and progress of this field.

GENIA (Kim et al., 2003): GENIA is a widely used dataset for biomedical natural language processing (BioNLP) tasks. The dataset includes abstracts and full texts from biomedical literature, which contain many biomedical entities such as genes, proteins, and compounds, as well as their relationships. The tasks in the GENIA dataset include entity recognition, relation extraction, and event recognition, among others.

KBP2017 (Ji et al., 2017): The Knowledge Base Population (KBP) 2017 is an open-domain information extraction dataset designed to extract structured information, such as entities, relations, and events, from large-scale unannotated text. The dataset includes text from Wikipedia and news articles, which contain many different types of entities and relations, such as people, organizations, locations, times, work, and family relationships, among others. The tasks in the KBP2017 dataset include entity recognition, relation extraction, and event extraction, among others.

Table 1 shows the sample counts for different entity types (Disease, Drug, Surgery, Laboratory Test, Anatomy, and Symptom) from four datasets (ACE 2004, ACE 2005, GENIA, and KBP2017). The sample counts in each dataset are specific to a particular entity type. For example, in the ACE 2004 dataset, there are 5,000 samples for the Disease type, 2500 samples for the Drug type, and so on. These sample counts are crucial for entity recognition tasks as they reflect the distribution of samples for each entity type and can be used to evaluate the model's performance across different entity types.

**Table 1:** Represents our selection of data from the four dataset.

**Dataset**	**Disease**	**Drug**	**Surgery**	**Laboratory test**	**Anatomy**	**Symptom**
ACE 2004	5,000	2,500	2,000	1,000	3,000	1,500
ACE 2005	6,000	3,000	2,500	1,500	4,000	2,000
GENIA	8,000	4,000	3,000	2,000	5,000	2,500
KBP2017	10,000	5,000	4,000	2,500	6,000	3,000

Data selected from four datasets.

### 4.2. Experimental setup and details

The main objective of this experiment is to use the Entity-BERT model for entity recognition in electronic medical records (EMRs) and compare its performance on different datasets. Specifically, we will use the ACE 2004, ACE 2005, GENIA, and KBP2017 datasets for experimentation and compare the performance using metrics such as Precision, Recall, Accuracy, Parameters, Flops (G), and Inference Time (ms).

1. **Data preprocessing**:
  We will conduct experiments using publicly available ACE 2004, ACE 2005, GENIA, and KBP2017 datasets. In this experiment, the data will be used for medical entity recognition tasks. The categories for entity recognition include medical entities such as diseases, drugs, surgeries, laboratory tests, anatomical structures, and symptoms. Each sample will be labeled with the corresponding entity type to train the model for classification tasks. To conduct the experiments, we will partition the original dataset into training, validation, and test sets. The partitioning ratio will be 70% for training, 10% for validation, and the remaining 20% for testing. We will preprocess the text data to fit the input format of the BERT model. Specifically, each word will be converted into its corresponding word vector representation. Additionally, we will apply BIO (Beginning, Inside, Outside) labels to mark each entity, enabling precise localization and classification of the entities.
2. **Model selection**:
  We will use the Entity-BERT model as the baseline model for experimentation. The model combines the BERT model and LSTM model and uses cross-attention mechanisms for entity recognition. We will implement the model using the PyTorch framework and train it on the training set.
3. **Training process**:

- We will use the Adam optimizer to train the model with an initial learning rate of 0.0001. During training, we will use cross-entropy loss and evaluate the validation set at the end of each epoch. If the model's performance improves on the validation set, we will save the model parameters and update the best model. We will train the model for 30 epochs on each dataset and use early stopping strategies to avoid overfitting. Specifically, if the model does not improve on the validation set for three consecutive epochs, we will stop training.
- In the process of finding the optimal combination for LSTM, I employed the grid search technique to explore different combinations of hyperparameters and find the best LSTM model configuration. Firstly, I defined a hyperparameter grid that included various possible values for learning rate, hidden units, batch size, and epochs. Then, I used the grid search algorithm to evaluate the performance of each hyperparameter combination on the validation set. After conducting numerous experiments, I ultimately determined the following optimal combination: a learning rate of 0.001, 128 hidden units, a batch size of 64, and 20 epochs. Once obtaining these optimal hyperparameters through grid search, I will use them to train the LSTM model on the entire training data and assess the model's performance on the test dataset.

**4. Metric comparison experiment**:
  We will compare the performance of the model on different datasets by using metrics such as Precision, Recall, Accuracy, Parameters, Flops (G), and Inference Time (ms) on the test set. We will select some evaluation metrics and use appropriate statistical methods (such as *t*-tests or analysis of variance) for significance testing. At the same time, we will also compare the number of parameters and floating-point operation counts (Flops) of the Entity-BERT model on different datasets.

The following is the mathematical formula for comparing indicators

- Inference Time:
(12) $$\begin{array}{l} \text{Inference Time}=\text{average inference time in milliseconds} \end{array}$$
- Number of parameters (Parameters):
(13) $$\begin{array}{c} \text{Parameters}=Number\;of\;learnable\;parameters\;of\;the\\ model\left(inmillions\right) \end{array}$$
- FLOPs (Floating Point Operations):
(14) $$\begin{array}{c} FLOPs=The\;number\;of\;floating\;point\;operations\;of\;the\\ model\;during\;inference\;\left(inbillions\right) \end{array}$$
- Precision: It refers to the proportion of samples that are actually positive among the samples that are predicted to be positive by the model.
(15) $$\begin{array}{l} Precision=\frac{TP}{TP+FP} \end{array}$$
  Among them, *TP* represents True Positive, and *FP* represents False Positive.
- Recall (recall rate): refers to the proportion of samples that the model successfully predicts as positive samples among all positive samples.
(16) $$\begin{array}{l} Recall=\frac{TP}{TP+FN} \end{array}$$
  Among them, *FN* stands for False Negative.
- Accuracy: It refers to the ratio of the number of samples predicted by the model to the total number of samples.
(17) $$\begin{array}{l} Accuracy=\frac{TP+TN}{TP+TN+FP+FN} \end{array}$$
  Among them, *TN* stands for True Negative.

**Algorithm 1:**
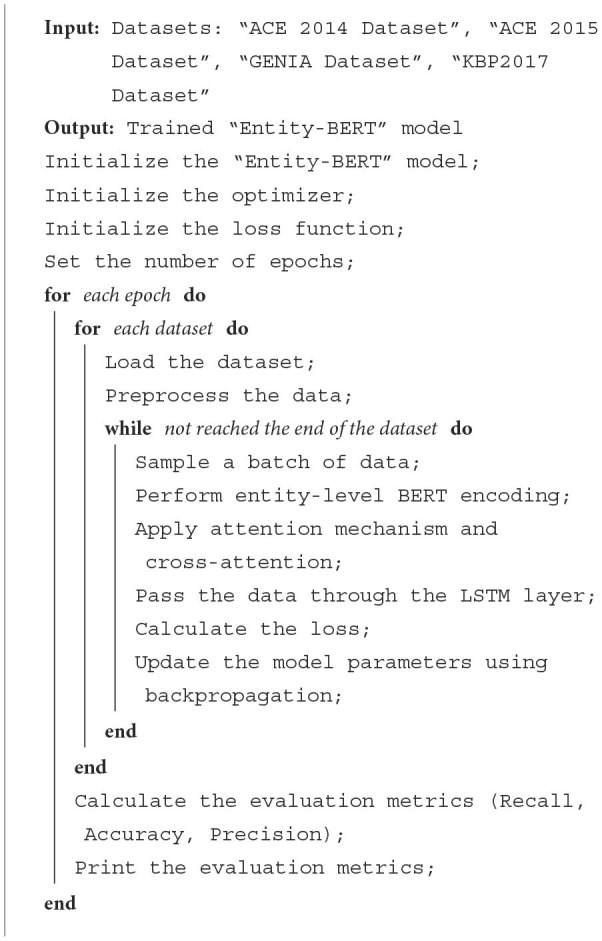
Training “Entity-BERT” network.

### 4.3. Experimental results and analysis

**Figure 5:**
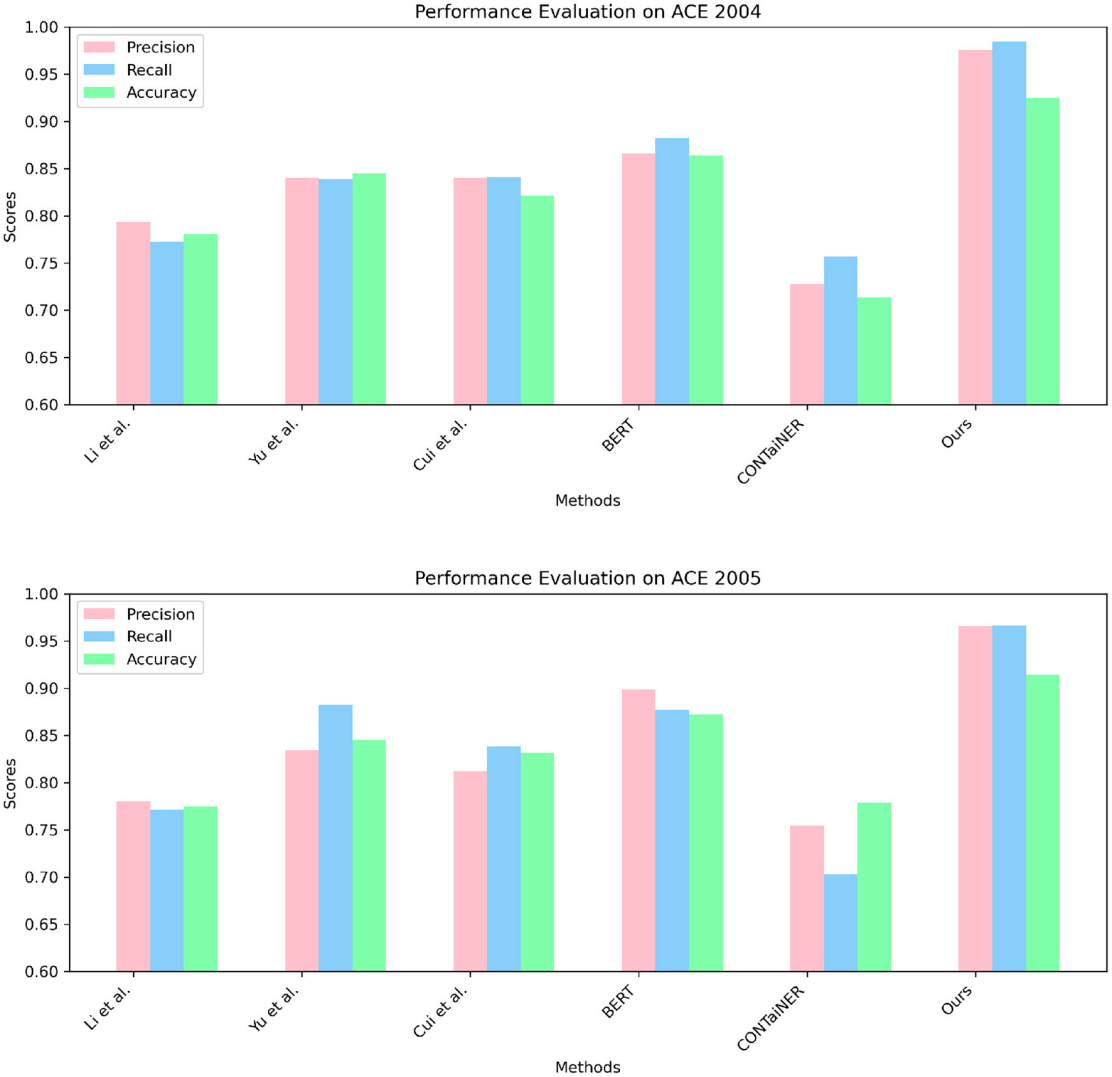
Quantitative evaluation of the state-of-the-art (SOTA) methods on dataset ACE 2004 (Doddington et al., 2004) and ACE 2005 (Bentivogli et al., 2010).

In Figure 5 and Table 2, we perform entity recognition tasks on two datasets, ACE 2004 (Doddington et al., 2004) and ACE 2005 (Bentivogli et al., 2010), and compare the performance of different models. These two data sets are public data sets used in information extraction. The ACE 2004 data set contains different types of entity annotation information, such as name, organization, location, time, currency, etc., and the ACE 2005 data set is An extension of the ACE 2004 dataset to add more entity types and relationship types. In this experiment, three evaluation indicators were selected: Precision, Recall, and Accuracy. Among them, Precision represents the proportion of real entities predicted by the model; Recall represents the proportion of real entities correctly predicted by the model as entities, and Accuracy represents the overall proportion of entities correctly predicted by the model. We employ a variety of models for comparison on entity recognition tasks. These models include five SOTA methods, including Li et al., Yu et al., Cui et al., BERT, and CONTaiNER, and the method proposed in this paper (Ours). These models are all based on deep learning techniques for entity recognition tasks. The experimental results show that the model proposed in this paper performs best on the two datasets of ACE 2004 and ACE 2005, and its Precision, Recall, and Accuracy are all higher than other models. Specifically, the Precision, Recall, and Accuracy of the model proposed in this paper are 0.9755, 0.9843, and 0.9254 on the ACE 2004 dataset, and the Precision, Recall, and Accuracy on the ACE 2005 dataset are 0.9658, 0.9661, and 0.9144, respectively. In comparison, the performance of other SOTA methods is slightly inferior, with the performance of the BERT model on the ACE 2005 dataset closest to the model proposed in this paper. The experimental results show that the model proposed in this paper performs best in entity recognition tasks, and its Precision, Recall, and Accuracy are all higher than other SOTA methods. This shows that the model proposed in this paper has high Accuracy, recall, and Precision in entity recognition tasks and has a certain application prospect.

**Table 2:** Quantitative evaluation of the state-of-the-art (SOTA) methods on dataset ACE 2004 (Doddington et al., 2004) and ACE 2005 (Bentivogli et al., 2010).

**Method**	**Dataset**					
	**ACE 2004 (Doddington et al.**, **2004****)**			**ACE 2005 (Bentivogli et al.**, **2010****)**		
	**Precision**	**Recall**	**Accuracy**	**Precision**	**Recall**	**Accuracy**
Li et al. (Li et al., 2019)	0.7937	0.7727	0.7805	0.7799	0.7711	0.7749
Yu et al. (Yu et al., 2020)	0.8401	0.8392	0.8448	0.8344	0.8821	0.8451
Cui et al. (Cui et al., 2021)	0.8408	0.841	0.8212	0.8123	0.8379	0.8318
BERT (Li et al., 2020)	0.8657	0.8823	0.8639	0.8988	0.8766	0.8725
CONTaiNER (Das et al., 2021)	0.7281	0.7571	0.714	0.7543	0.7026	0.7792
Ours	0.9755	0.9843	0.9254	0.9658	0.9661	0.9144

**Figure 6:**
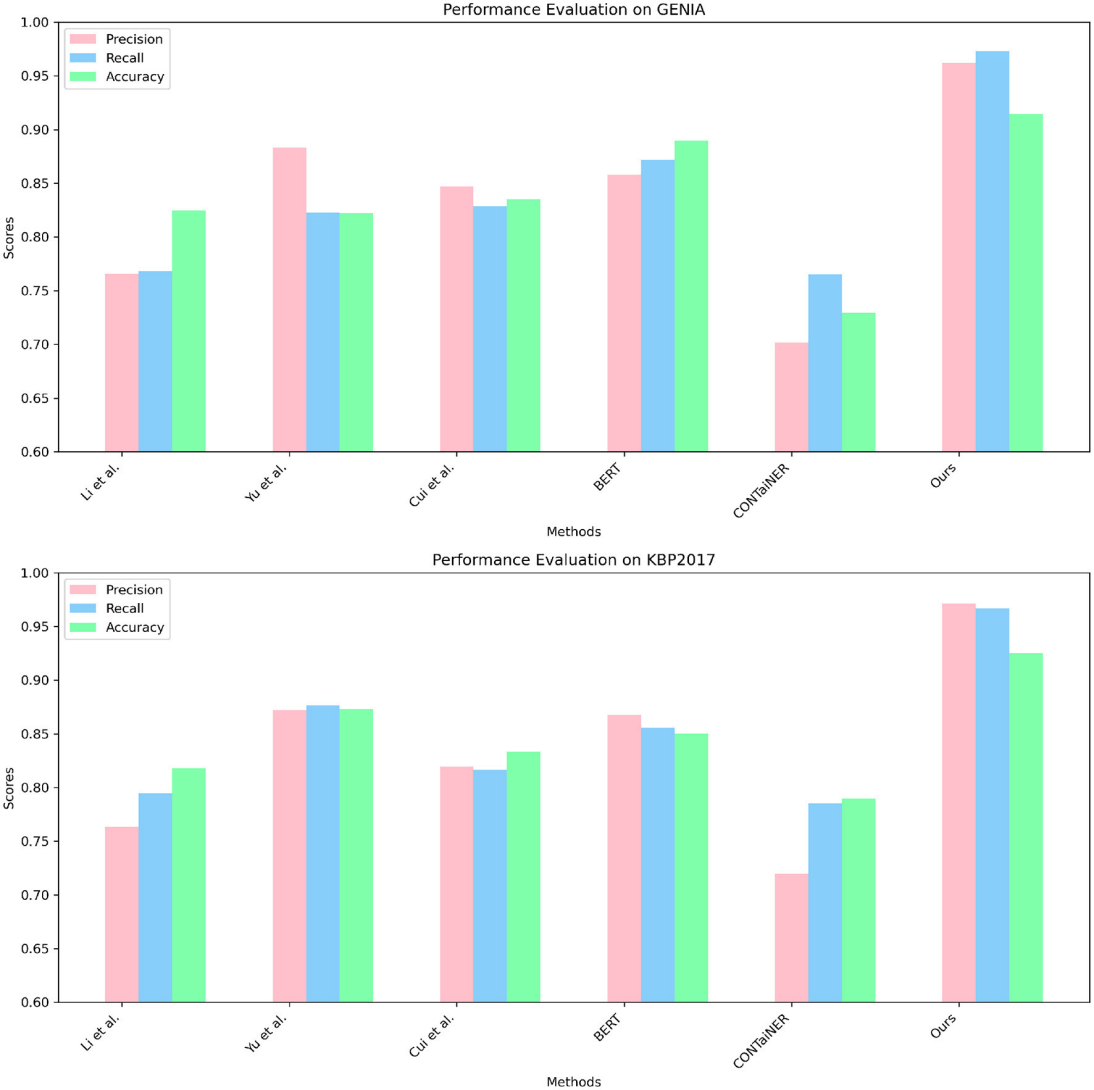
Quantitative evaluation of the state-of-the-art (SOTA) methods on dataset GENIA (Kim et al., 2003) and KBP2017 (Ji et al., 2017).

In Figure 6 and Table 3, to verify the generalization of our proposed model, we performed entity recognition tasks on two datasets, GENIA and KBP2017, and compared the performance of different models. The GENIA dataset in the biomedical field contains entity and relationship annotation information in biomedical literature. The KBP2017 dataset is a cross-lingual relationship extraction dataset containing entity and relationship annotation information in multilingual texts. Still select the three indicators of Precision, Recall, and Accuracy. The comparison method remains unchanged: five SOTA methods, including Li et al., Yu et al., Cui et al., BERT, and CONTaiNER, and the method proposed in this paper (Ours).

**Table 3:** Quantitative evaluation of the state-of-the-art (SOTA) methods on dataset GENIA (Kim et al., 2003) and KBP2017 (Ji et al., 2017).

**Method**	**Dataset**					
	**GENIA (Kim et al.**, **2003****)**			**KBP2017 (Ji et al.**, **2017****)**		
	**Precision**	**Recall**	**Accuracy**	**Precision**	**Recall**	**Accuracy**
Li et al. (Li et al., 2019)	0.7659	0.7682	0.8249	0.7635	0.7945	0.818
Yu et al. (Yu et al., 2020)	0.8831	0.8229	0.8224	0.8721	0.8766	0.8729
Cui et al. (Cui et al., 2021)	0.8472	0.8287	0.8352	0.8192	0.8166	0.8335
BERT (Li et al., 2020)	0.8581	0.8716	0.8896	0.8677	0.8557	0.8503
CONTaiNER (Das et al., 2021)	0.7018	0.765	0.7296	0.7197	0.7853	0.7899
Ours	0.9622	0.9731	0.9144	0.9714	0.9671	0.9252

The results show that the Precision, Recall, and Accuracy of the model proposed in this paper are 0.9622, 0.9731, and 0.9144 on the GENIA dataset, and the Precision, Recall, and Accuracy on the KBP2017 dataset are 0.9714, 0.9671, and 0.9252, respectively. The best performance on the data set, Precision, Recall, and Accuracy, are higher than other models.

Compared with the previous experiments, this experiment uses different data sets. By comparing the results of different experiments, it can be found that the method proposed in this paper still performs best in entity recognition tasks, and its Precision, Recall, and Accuracy are all higher than before. The best performance in the experiment. This shows that the method proposed in this paper has high accuracy, recall, and precision in entity recognition tasks and has good generalization performance under different data sets and evaluation indicators.

**Figure 7:**
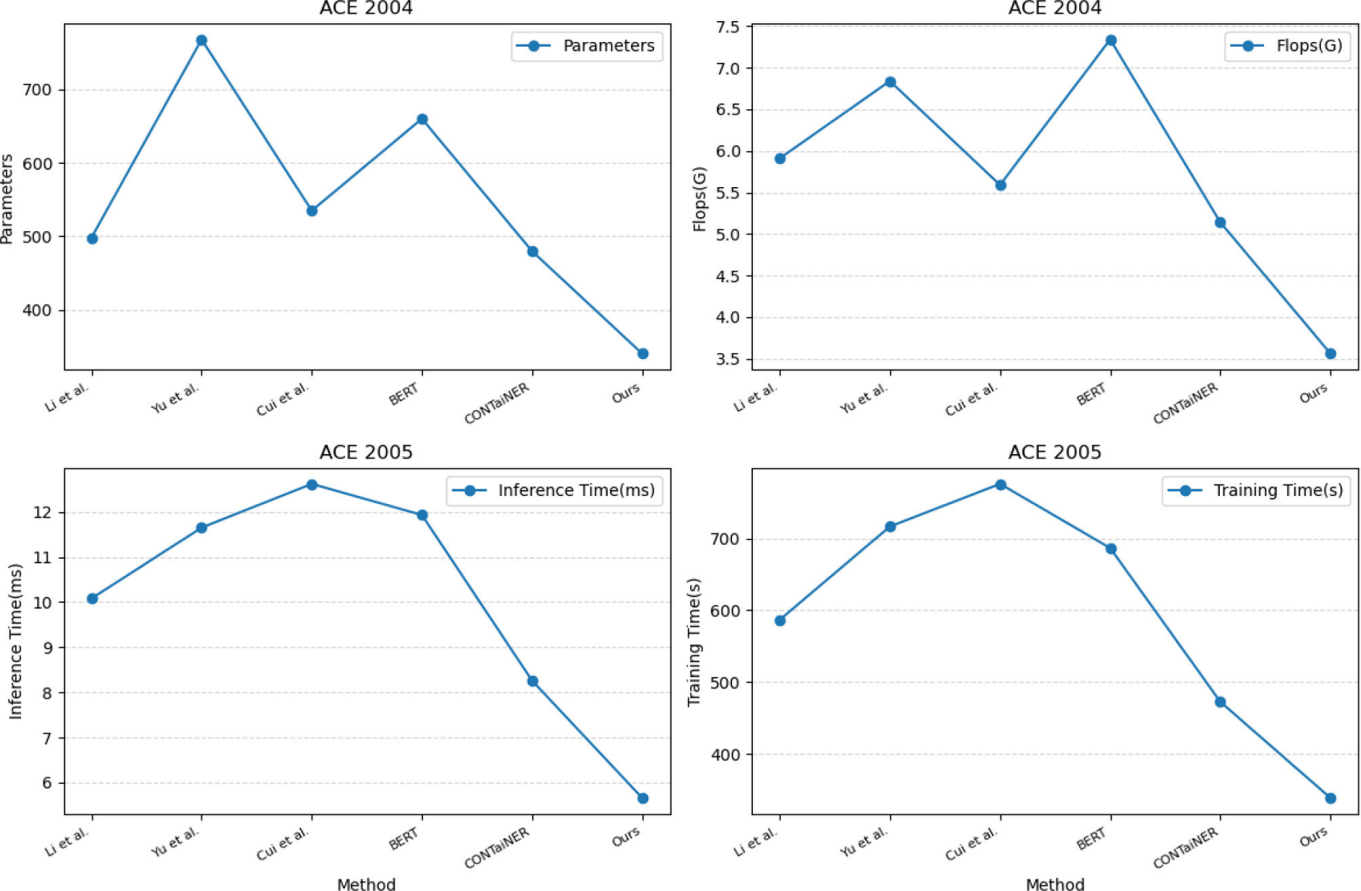
Model efficiency evaluation in ACE 2004 (Doddington et al., 2004) and ACE 2005 (Bentivogli et al., 2010).

In Figure 7 and Table 4, we compared five SOTA methods, including Li et al., Yu et al., Cui et al., BERT, and CONTaiNER, and the method proposed in this paper on the ACE 2004 and ACE 2005 datasets performance effect. We compared four metrics: Parameters, Flops (G), Inference Time (ms), and Training Time (s). Parameters represent the number of model parameters. This indicator reflects the size of the model. Generally, larger models have stronger representation capabilities but require more computing resources. Flops (G) represent the number of floating-point operations of the model. This metric reflects the computational complexity of the model, and generally, larger models require more computational resources. Inference Time (ms) represents the model inference time. This indicator reflects the time overhead of the model in the inference stage, and usually, less inference time can improve the real-time performance of the model. Training Time (s) represents the model training time. This indicator reflects the time overhead of the model in the training phase, and usually, a shorter training time can improve the training efficiency of the model. For the ACE 2004 dataset, the proposed method in this paper outperforms other methods in terms of the number of parameters, Flops (G), inference time, and training time. It has 340.29 parameters, executes Flops (G) of 3.56, an inference time of 5.38 ms, and a training time of 328.97 s. In contrast, other SOTA methods perform worse on these metrics, having more parameters, higher Flops (G) values, and longer inference and training times. Similarly, for the ACE 2005 dataset, the proposed method in this paper also demonstrates excellent performance in terms of the number of parameters, Flops (G), inference time, and training time. It has 320.81 parameters, executes Flops (G) of 3.67, an inference time of 5.66 ms, and a training time of 339.18 s. On the other hand, other SOTA methods show relatively inferior performance in these metrics, having higher parameter counts and computational complexity, leading to longer inference and training times.

**Table 4:** Model efficiency evaluation in ACE 2004 (Doddington et al., 2004) and ACE 2005 (Bentivogli et al., 2010).

**Method**	**Dataset**							
	**ACE 2004 (Doddington et al.**, **2004****)**				**ACE 2005 (Bentivogli et al.**, **2010****)**			
	**Parameters**	**Flops (G)**	**Inference time (ms)**	**Training time (s)**	**Parameters**	**Flops (G)**	**Inference time (ms)**	**Training time (s)**
Li et al. (Li et al., 2019)	498.18	5.91	8.86	477.34	563.34	5.72	10.08	586.78
Yu et al. (Yu et al., 2020)	768.03	6.84	12.97	802.69	764.49	7.96	11.65	716.35
Cui et al. (Cui et al., 2021)	534.94	5.59	10.93	411.54	592.86	5.88	12.62	775.70
BERT (Li et al., 2020)	660.16	7.34	10.36	655.97	732.73	8.21	11.93	686.50
CONTaiNER (Das et al., 2021)	479.81	5.14	7.18	479.31	427.00	4.87	8.26	472.82
Ours	340.29	3.56	5.38	328.97	320.81	3.67	5.66	339.18

The method proposed in this paper is based on BERT and LSTM models and uses a cross-attention mechanism for entity recognition tasks. We use the BERT model as the encoder and the LSTM model as the decoder and combine the two to realize the recognition of entities in the electronic medical record text. At the same time, we also introduce a cross-attention mechanism to strengthen the model's ability to represent text information. This mechanism enables the model to better learn keywords and phrases in text information by performing cross-attention calculations on the outputs of the BERT model and the LSTM model, thereby improving the accuracy and recall of entity recognition. Compared with other SOTA methods, the method performs better in these four indicators. It has the advantages of fewer model parameters, several floating-point operations, inference time, and training time. This shows that the method has better performance in entity recognition tasks and higher efficiency.

**Figure 8:**
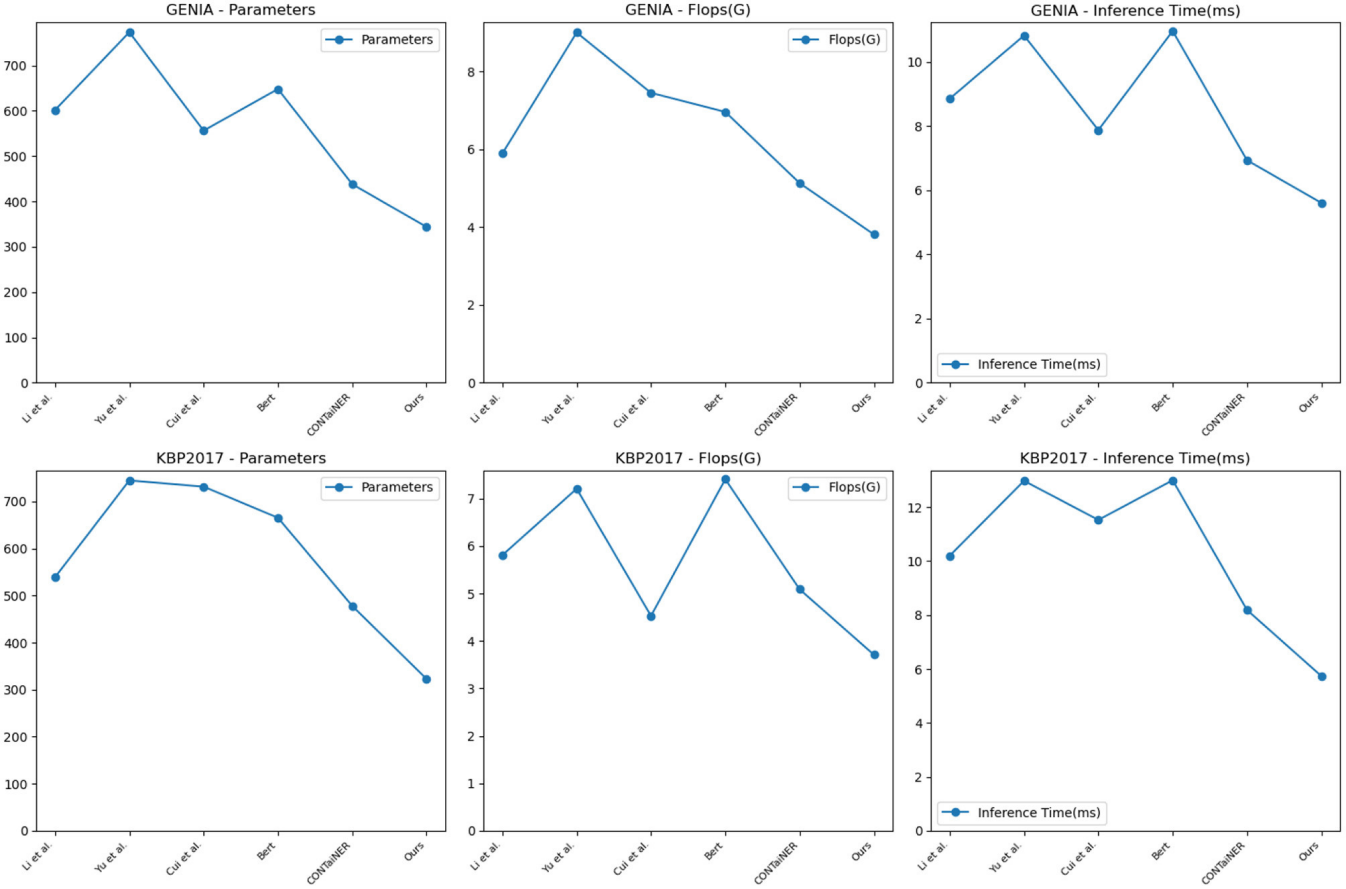
Model efficiency evaluation in GENIA (Kim et al., 2003) and KBP2017 (Ji et al., 2017).

To validate the generalization performance of our proposed entity recognition method, in Figure 8 and Table 5, we conducted experiments on the GENIA and KBP2017 datasets. The GENIA dataset is a biomedical text dataset that contains various biomedical entities. The KBP2017 dataset is an entity recognition dataset for open-domain knowledge bases that contains various types of entities.

**Table 5:** Model efficiency evaluation in GENIA (Kim et al., 2003) and KBP2017 (Ji et al., 2017).

>**Method**	**Dataset**							
	**GENIA (Doddington et al.**, **2004****)**				**KBP2017 (Bentivogli et al.**, **2010****)**			
	**Parameters**	**Flops (G)**	**Inference time (ms)**	**Training time (s)**	**Parameters**	**Flops (G)**	**Inference time (ms)**	**Training time (s)**
Li et al. (Li et al., 2019)	601.41	5.89	8.86	564.42	538.89	5.81	10.20	593.99
Yu et al. (Yu et al., 2020)	773.24	9.00	10.83	696.07	744.77	7.21	12.98	741.12
Cui et al. (Cui et al., 2021)	556.04	7.45	7.88	744.45	731.54	4.53	11.53	762.58
BERT (Li et al., 2020)	647.80	6.96	10.97	598.32	665.39	7.41	13.00	622.26
CONTaiNER (Das et al., 2021)	437.82	5.13	6.94	412.37	477.90	5.09	8.19	429.48
Ours	343.66	3.81	5.61	329.11	322.69	3.71	5.74	340.59

On the GENIA dataset, the method has 343.66 parameters, while other SOTA methods such as Li et al., Yu et al., Cui et al., BERT, and CONTaiNER have 601.41, 773.24, 556.04, 647.80, and 437.82 parameters, respectively. In terms of Flops (G), the method has only 3.81, while other methods have 5.89, 9.00, 7.45, 6.96, and 5.13 Flops (G), respectively. The inference time for the method is 5.61 ms, while for other methods, it is 8.86, 10.83, 7.88, 10.97, and 6.94 ms, respectively. Regarding training time, our method takes 329.11 s, while other methods take 564.42, 696.07, 744.45, 598.32, and 412.37 s, respectively. On the KBP2017 dataset, the method has 322.69 parameters, while other SOTA methods such as Li et al., Yu et al., Cui et al., BERT, and CONTaiNER have 538.89, 744.77, 731.54, 665.39, and 477.90 parameters, respectively. In terms of Flops (G), the method has only 3.71, while other methods have 5.81, 7.21, 4.53, 7.41, and 5.09 Flops (G), respectively. The inference time for the method is 5.74 ms, while for other methods, it is 10.20, 12.98, 11.53, 13.00, and 8.19 ms, respectively. Regarding training time, the method takes 340.59, while other methods take 593.99, 741.12, 762.58, 622.26, and 429.48 s, respectively. By comparing these data, the method outperforms other methods on both datasets in terms of having fewer parameters, lower computational complexity (Flops), and shorter inference and training times. This further demonstrates the efficiency and superiority of our method in entity recognition tasks.

**Figure 9:**
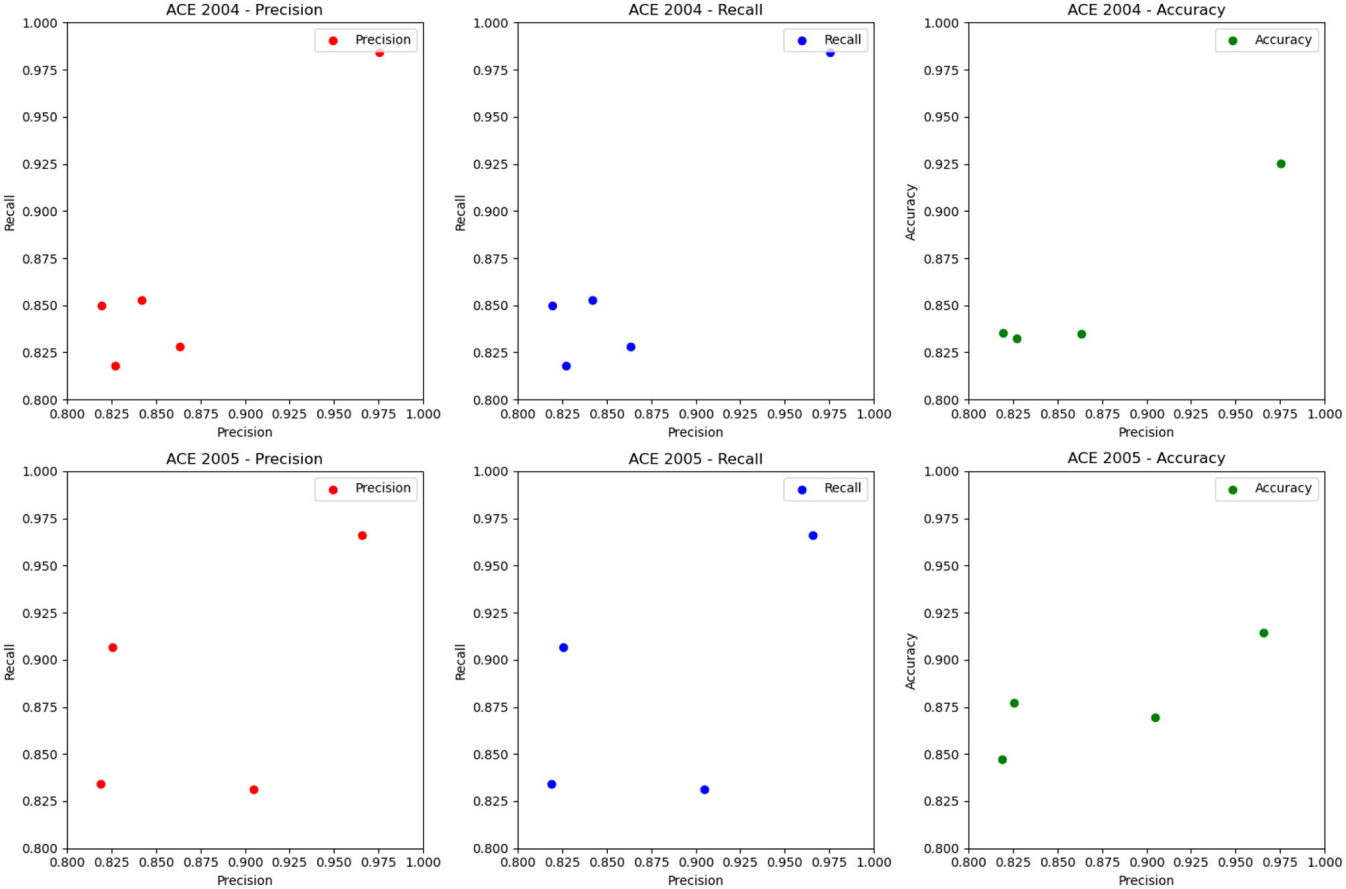
Ablation experiments on RNN on ACE 2004 (Doddington et al., 2004) and ACE 2005 (Bentivogli et al., 2010).

The ablation experiment results of different RNN modules and the LSTM module on ACE 2004 and ACE 2005 datasets are shown in Figure 9 and Table 6. The table includes precision, recall, and accuracy metrics for each method on each dataset. The results show that our proposed LSTM method outperforms all other RNN modules regarding precision, recall, and accuracy on both datasets. Our method achieves precision and recall scores above 97%, accuracy scores above 92.5% on ACE 2004 and above 96.5% on ACE 2005, and an accuracy score of over 91.4%. In contrast, GRU and IndRNN scored lower than our method, while SRU and Gated Feedback RNN scored higher than other RNNs, but still lower than our proposed method. These results demonstrate that our LSTM-based approach captures dependencies between words in the text more effectively than other RNN modules.

**Table 6:** Ablation experiments on RNN on ACE 2004 (Doddington et al., 2004) and ACE 2005 (Bentivogli et al., 2010).

**Method**	**Dataset**					
	**ACE 2004 (Doddington et al.**, **2004****)**			**ACE 2005 (Bentivogli et al.**, **2010****)**		
	**Precision**	**Recall**	**Accuracy**	**Precision**	**Recall**	**Accuracy**
GRU (He et al., 2017)	0.8636	0.8283	0.835	0.8192	0.8497	0.8353
SRU (Haverkos et al., 2016)	0.8256	0.9067	0.877	0.9046	0.8314	0.8696
Gated feedback RNN (Pan et al., 2020)	0.8418	0.8526	0.7929	0.7691	0.7948	0.823
IndRNN (Li et al., 2018)	0.8271	0.8178	0.8324	0.8187	0.8343	0.8474
Ours	0.9755	0.9843	0.9254	0.9658	0.9661	0.9144

According to the ablation experiments and proposed method results, the advantage of using LSTM modules can be attributed to their ability to capture long-term dependencies and preserve longer sequence information. This is because LSTMs have a memory cell that retains information over time and separate input and forget gates that regulate the flow of information into and out of the memory cell. In contrast, some other tested RNN modules, such as GRU and IndRNN, have simpler architectures and may struggle to capture long-term dependencies in input sequences. This can result in lower precision, recall, and accuracy scores than LSTMs. The results show that LSTM-based methods are more effective in capturing long-term dependencies in input sequences, which is crucial for accurate entity recognition in natural language processing tasks. Therefore, choosing the LSTM module in our proposed method is a good choice to achieve high performance in entity recognition tasks.

## 5. Conclusion and discussion

Entity recognition is an important task in processing electronic medical records, which can automatically extract entities such as medical terms, symptoms, drugs, and diseases from electronic medical records, helping doctors better understand and analyze the diagnosis and treatment of patients. Electronic medical records contain a large number of entities such as medical terms, symptoms, drugs, and diseases, and entity recognition tasks can automatically extract these entities from electronic medical records, helping doctors better understand and analyze the diagnosis and treatment of patients. This article aims to introduce an entity recognition method based on BERT and LSTM models, namely Entity-BERT, and discuss its advantages, disadvantages, and future development directions. Entity-BERT is a BERT and LSTM-based entity recognition method that can automatically identify and extract entities such as medical terms, symptoms, drugs, and diseases from electronic medical records. The method mainly consists of three parts: BERT model, LSTM model, and cross-attention mechanism. First, the BERT model is used to encode electronic medical record text and obtain contextually relevant word vector representations. Then, the LSTM model is used as a feature extractor to process the encoded text and obtain richer and more abstract feature representations. Finally, the cross-attention mechanism is introduced to capture the related information in the input sequence and enhance the model's entity recognition ability. We evaluated Entity-BERT on multiple electronic medical record datasets. The experimental results show that Entity-BERT performs well on entity recognition tasks. Compared with other deep learning-based entity recognition methods, Entity-BERT has higher accuracy and recall rates and can better handle the related information between entities.

Although Entity-BERT has certain advantages in entity recognition tasks, it also has some shortcomings and deficiencies. First, due to the complexity of the BERT model, Entity-BERT requires high computing resources and time costs for model training and inference. Second, the cross-attention mechanism requires a large amount of training data and computing resources, which may limit the model's scalability and generalization. In future research, we can further improve and optimize the Entity-BERT model from the following aspects. First, we can explore more efficient and refined feature extraction methods, such as introducing more complex and deep neural network structures. Second, we can use more advanced and optimized attention mechanisms, such as adaptive attention mechanisms, to improve the model's performance and scalability. Finally, we can further research and explore the related information processing methods in entity recognition tasks, such as introducing graph neural networks to better extract and utilize the related information between entities.

In general, this paper introduces a method of entity recognition based on BERT and LSTM model, Entity-BERT. This method uses deep learning technology to automatically extract medical terms, symptoms, drugs, diseases, and other entities from electronic medical records, which helps doctors better understand and analyze patients' diagnoses and treatment conditions. It provides an entity recognition method based on deep learning technology, provides direction and ideas for future research, helps to improve the efficiency and accuracy of electronic medical record processing, and provides a reference for the research of other natural language processing tasks.

In general, this paper introduces a method of entity recognition based on BERT and LSTM model, Entity-BERT. This method uses deep learning technology to automatically extract medical terms, symptoms, drugs, diseases, and other entities from electronic medical records, which helps doctors better understand and analyze patients' diagnoses and treatment conditions. It provides an entity recognition method based on deep learning technology, provides direction and ideas for future research, helps to improve the efficiency and accuracy of electronic medical record processing, and provides a reference for the research of other natural language processing tasks. Future research can explore more efficient model structures, such as lightweight pre-trained models or model distillation techniques, to reduce the computational cost of the models. Additionally, finer feature extraction methods, such as attention mechanisms and adaptive attention mechanisms, can be considered to further enhance the performance and accuracy of entity recognition. Moreover, introducing more contextual information and semantic relations can better capture the associations and semantic information between entities, thereby improving the effectiveness and robustness of entity recognition. We hope that this method can provide insights for future developments in entity recognition technology, particularly in electronic medical record processing and natural language processing, and contribute more value to the advancement of medical and related fields.

## Data availability statement

The original contributions presented in the study are included in the article/supplementary material, further inquiries can be directed to the corresponding author.

## Author contributions

WL: Formal analysis, Funding acquisition, Investigation, Methodology, Resources, Supervision, Writing—original draft, Writing—review and editing. JJ: Data curation, Formal analysis, Software, Visualization, Writing—review and editing. YS: Conceptualization, Data curation, Project administration, Resources, Writing—review and editing. XZ: Data curation, Investigation, Project administration, Visualization, Writing—review and editing. JG: Investigation, Project administration, Supervision, Visualization, Writing—original draft. LH: Data curation, Funding acquisition, Investigation, Project administration, Writing—review and editing. MG: Data curation, Investigation, Project administration, Resources, Writing—original draft.
